# Application of “Behind the Barriers” Model at Neighbourhood Scale to Improve Water Management under Multi-Risks Scenarios: A Case Study in Lyon, France

**DOI:** 10.3390/ijerph20032587

**Published:** 2023-01-31

**Authors:** Bruno Barroca, Maria Fabrizia Clemente, Zhuyu Yang

**Affiliations:** 1 Lab’urba, Université Gustave Eiffel, 77420 Champs-sur-Marne, France; 2 Department of Architecture, University of Naples Federico II, 80134 Naples, Italy; 3 LATTS, UMR CNRS 8134 Université Gustave Eiffel/Ecole des Ponts ParisTech, 77420 Marne la Vallee, France

**Keywords:** water management, “Behind the Barriers” model, resilience, urban water cycle, water balance, water management, performance assessment, neighbourhood, Part-Dieu

## Abstract

In modern urban areas, water management highly depends on the socio-ecological urban water cycle (UWC) that heavily relies on water infrastructures. However, increasing water-related hazards, natural and/or human-based, makes it difficult to balance water resources in the socio-ecological UWC. In the last decade, urban infrastructure resilience has rapidly become a popular topic in disaster risk management and inspired many studies and operational approaches. Among these theories and methods, the “Behind the Barriers” model (BB model), developed by Barroca and Serre in 2013, is considered a theory that allows effective and comprehensive analysis of urban infrastructure resilience through cognitive, functional, correlative, and organisational dimensions. Moreover, this analysis can be a reference to develop actions that improve infrastructure resilience under critical scenarios. Therefore, this study aims to study resilience design actions based on the BB model to achieve socio-ecological water balance and assess the performance of these actions. The study focuses on water management on a neighbourhood scale, which is considered the essential urban unit to study and improve the resilience of critical infrastructures, such as water services. The Part-Dieu neighbourhood in Lyon, France is selected as a case study, and it highlights the need to develop indicators to assess the performance of implemented actions in a structural and global resilience framework, to understand urban systems as complex and dynamic systems to provide decision support, and to strengthen crisis prevention and management perspectives in a dynamic approach.

## 1. Introduction

Environmental changes, combined with the concentration of property and persons in urban territories, foretell devastating events in the coming years. The consequences of the frequency and severity of climate extremes will lead directly to more intense droughts alternating with heavier rain and snowfalls. Indirectly, global changes can produce several health risks, disturbances in urban activities, and ecological imbalances [1,2,3]. Given the fact that environmental changes also imply an ever-increasing uncertainty in risk assessment, new risk management strategies need to be developed for anticipating scenarios that probabilistic models judge to be extreme or rare at present [4,5].

According to a recent report by the United Nations, 55% of the world’s population lives in urban areas, a proportion that is expected to increase to 68% by 2050 [6]. Water constitutes a basic element of human and urban civilisations. The discussion on urban water resources is becoming more and more indispensable because of its crucial role in future human development [7,8]. Climate change, growth and rapid urbanisation, and sudden changes in water demand are making urban water problems severe; moreover, the extreme weather risks following climate change, such as droughts, floods, and less predictable rainfall and urban heating, increase the vulnerability of urban water management [9,10,11,12,13].

As also highlighted in the latest IPCC AR6 report, the problem of “water scarcity” will increase in small islands and the Mediterranean region, while rainfall will increase in northern Europe and decrease in southern Europe causing major challenges for irrigation, hydropower, ecosystems, and urban settlements [3]. In Europe, about 11% of the population is at risk of water scarcity and unsafe water supply, conditions that contribute to the death of 14 people every day. In this context, the COVID-19 pandemic has added further pressure on the global water sector, which is already under severe pressure from climate change, population growth, and inadequate infrastructures [14,15].

At the urban level, water performs multiple functions through a complex cycle known as the urban water cycle (UWC). The concept of water cycle came from the ecologic hydrologic cycle and evolved into a chain of urban water services to the urban population, including water supply, drainage, wastewater collection, and water management. The UWC system highly relies on many technical infrastructures, for example: water supply infrastructures to import large quantities of water to users, drainage infrastructure to create a runoff collection and transportation system to reduce the risk of flooding and surface water ponding, or sewage treatment infrastructures to treat contaminants from municipal wastewater. All urban functions depend directly on water resources, and urban infrastructures are interdependent and essential for city performances [16,17,18].

In this scenario, the UWC is, therefore, a key strategy for sustainable and resilient water management. Inefficient water management leads to numerous urban risks, such as water scarcity, urban flooding, or drought. Sustainable and resilient systems need to be envisaged to address urban water-related risks, and alternative sources need to be evaluated to make the infrastructure smarter and more resilient [19,20,21]. It is necessary to move from linear models to circular models of water management [22].

In the very recent past, the notion of vulnerability has formed the dominant paradigm for risk and disaster analysis; over the last few years, resilience appears to be a concept that is a bearer of hope. The vulnerability concept focuses on how to keep cities protected from hazards, while the resilience concept considers accepting hazards into cities, but transforms them into a non-risk factor [23,24,25,26]. If urban resilience can be defined as “the ability of an urban system-and all its constituent socio-ecological and socio-technical networks across temporal and spatial scales-to maintain or rapidly return to desired functions in the face of a disturbance, to adapt to change, and to quickly transform systems that limit current or future adaptive capacity” [27], water resilience can be defined as the role water plays in safeguarding and sustaining the state of equilibrium of socio-ecological systems. Maintaining this balance is fundamental to ecosystems and biomes, the stability of regional weather and climate systems, the stable water supply required by societies, and the state of the biosphere and earth system [28,29,30]. This condition at a local scale, within the framework of the UWC, is defined as water balance. Water balance can thus be considered an ideal resilient state achieved through local water management, consisting of the balanced relation between water storage, water inflow, and water outflow [31,32,33].

The purpose of this article is to study the resilience conditions of the water balance state, on a neighbourhood scale, by applying the “Behind the Barriers” conceptual model (BB model), developed by Barroca and Serre in 2013 [34]. This model suggests overcoming the barriers of the traditional thinking of urban disaster risk management, accepting the existence of hazards, and focusing on making urban systems more adaptive to them rather than resisting them with barriers. In the model, urban systems are conceptualised as complex systems, and long-term impacts and connections with the external environment are considered to overcome the barriers of temporal, geographic, and/or dimensional perimeters.

In this study, water balance is identified as a key condition for city performance under pressure from both climatic stresses such as flooding or water scarcity and/or human stresses such as the COVID-19 pandemic. To achieve the study goal, the background related to urban resilience and water management and the “Behind the Barriers” model (BB model) is presented in Section 2. The methods of this study are then described in Section 3. Section 4 shows the application of the model to the case study of Lyon, Part-Dieu neighbourhood and, specifically, to the new project of Garibaldi Street. In Section 5, the results are discussed highlighting the importance of “going beyond barriers” (physical and temporal) to design resilient systems. Finally, the conclusions outline future research perspectives (Section 6).

## 2. Background

### 2.1. Urban Resilience and Water Management

Following the growing impacts of climate change, urban hazards concerning water systems are various and complex. In urban areas, increased precipitation or malfunction of drainage systems can cause flooding that leads to direct damages or potential indirect impacts on different urban sectors, such as the economy, citizens’ security, critical infrastructures, etc. Conversely, a decrease in rainy precipitation may cause water shortages for households, industries, and services; reduced availability of irrigation water; and even reduced energy supply performance [35].

Studying urban resilience poses the question of the pertinent spatial as well as temporal scales. Certainly, resilience is a concept that has advanced concerning the dynamic development of complex adaptive systems with interactions across temporal and spatial scales [36]. According to the perspective of complex adaptive systems, a first process corresponds to urban system resilience following a disturbance that caused damages at a local urban scale, and it seeks to improve the system’s capacity to respond to disturbances; resilience is here considered as a property, an inherent quality of a system. On the other hand, on a more global scale, considering the long-term impacts of disturbances, urban resilience can be defined as a process that leads to a condition of resilience. Consequently, the goal is to respond to short-term challenges through actions at the local scale, but also to promote long-term mechanisms to respond positively to possible future stresses [37,38,39].

Going across national urban levels (territory/city) to local urban levels (neighbourhood/building) allows identifying methods and tools focusing on urban water systems that assess the impacts of climate change, vulnerability, and adaptation options [40,41]. Some initiatives taking place at the territory and/or city level aim to operationalise urban resilience in a holistic perspective, integrating different dimensions of urban systems which involve technical and organisational aspects [42,43]. Other research suggests operationalising urban resilience in a more specific way, focusing on a specific urban dimension and/or component such as urban planning and the design of water systems [44] or urban landscapes [45]. Considering the physical urban dimension and focusing on technical aspects, some other studies have evolved to implement strategies based on mixed approaches to improve protection and adaptation; these studies are mostly focused on urban networks and infrastructures and consider structural, functional, and organisational aspects [46,47,48]. At a more local urban scale, in some instances, urban resilience analyses are based on buildings [49,50].

To assess a resilient state of water balance, it is necessary to consider the neighbourhood spatial scale as these systems involve multiple scales including users, institutions, technologies, and ecosystems [51,52]. A neighbourhood can be considered a social, economic, and physical/technical system; the study of resilience, therefore, must involve, in a holistic perspective, these different urban dimensions [37]. In this contribution, only the built environment is considered and thus only the physical/technical aspects of the neighbourhood. Focusing on the physical dimension and using a systemic approach, we can then identify a system whose main urban components include buildings, transport networks, and infrastructures, among others.

Looking at the state of the art in the reference literature, many studies address urban water services in operational guidelines aimed at disaster risk reduction and resilience, but fewer consider resilience as an operationalised concept [27,52,53,54,55]. Hence, in Section 3 the “Behind the Barriers” conceptual model will be proposed and applied at the neighbourhood scale to provide a reference framework to study, analyse, and improve the resilience of urban neighbourhoods, specifically focused on urban water management.

### 2.2. The “Behind the Barriers” Model

The comprehensive transverse approach, which is the distinctive feature of a project rationale, is fundamental when we look at the question of risk management. In practice, for “territory risks”, i.e., risks for which it is possible to set and circumscribe the area under threat (which is not the case in certain diffuse hazards such as epidemics or certain types of pollution), the innovation engine generally results from a political determination to consider all the different elements, especially those concerning the definition of resilience objectives. However, operational implementation, and even more the efficiency of the systems installed, presuppose possessing precise knowledge and associating all the players involved in planning and risk management.

**Figure 1:**
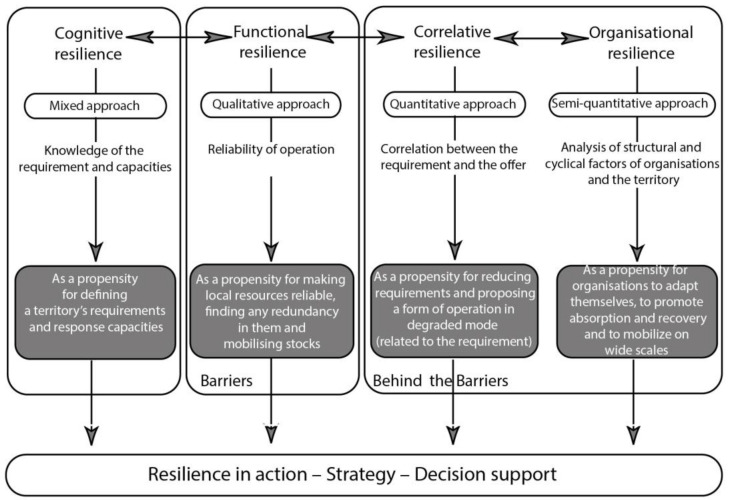
“Behind the Barriers” conceptual model. Source: Barroca and Serre [34].

The recent introduction of the resilience factor in the field of natural and technological risks is considered to provide new perspectives in the prevention and management of any associated events. Following the “Behind the Barriers” model (BB model), which was developed in 2013 [34] and applied in several studies [56,57], four dimensions of resilience have been identified [58,59] (see Figure 1):

- A cognitive dimension refers to knowledge, awareness, and the identification of resilience by the persons concerned. This dimension incorporates the assessment of resilience factors as well as the methods and tools that can be used for measuring them. How can the resilience of a technical urban system, a territory, or a natural environment be assessed? Does taking account of resilience change our way of observing, measuring, analysing, or representing urban systems?
- A functional dimension specific to material objects and technical urban systems forming the territory. Guaranteeing maintenance of service by the most important infrastructures corresponds to a type of resilience called “functional resilience”. Functional resilience is applied by carrying out reliability and operational dependability actions. To what extent do present approaches to risk management applied in urban environments need to be rethought and modified for taking into account resilience?
- A correlative dimension that recognises that service and utilisation form a whole whose different sections are interconnected together. None of these parts can vary without the others varying as well, and a system’s resilience can only exist when the way it operates in a degraded mode is acceptable in all risk periods. What can the effects of this dimension be on design, ways of development, operating conditions, and management of technical networks?
- A territorial/organisational dimension that raises the question of the persons involved (public and private players, populations, etc.) and the strategies that contribute to improving resilience.

A resilient system should have capabilities in these four dimensions.

The applications of the BB model to resilience analysis are wide and various, as it is a multidisciplinary, transversal resilience theory aiming at adaptation capacity. Gonzva [56] considers the BB model corresponding to a synthetic structuring of resilience in urban engineering. Gonzva and Barroca [57] assess the four capabilities of the BB model for a rail transport system, which has multiple possible implementing actions to improve resilience.

In this study, since resilience is considered operational, the BB model could be a basis for planning implementing actions that improve the resilience of water balance. This idea requires that the implementing actions should be designed to target the competencies of the four dimensions (cognitive, functional, correlative, and organisational). The performance or benefit of implemented actions could be assessed through indicators. The above acceptance is advanced as a hypothesis of this study.

## 3. Methods

### 3.1. The Water Balance

In water management, the concept of water resilience is related to the condition of “Water balance”, which involves the hydrologic system as well as the natural water and the socio-economic water systems [33,60].

The first step of water balance requires the assessment of the freshwater resource: the natural water balance. According to the European Commission [33], in the case of water balance the inflow has to be equal to the water outflow plus the changes in water storage (Equation (1)):

Inflow = Outflow ± Changes in Storage(1)

This condition means that the natural water inflow (ni) needs to be equal to natural water outflow (no) ± natural water storage (ns) (Equation (2)):

ni = no ± ns(2)

Considering the sub-indicators, natural water inflow is related to the precipitation water (ni), converted in natural water outflow associated with water evaporation (surface, interception, and transpiration) (no_1_) and/or water runoff (surface, subsurface, or groundwater) (no_2_) ± associated storage or changes in storage (ns) (Equation (3)):

ni = no_1_ + no_2_ ± ns (3)

Human activities influence the hydrological balance by using or discarding a certain volume of water or modifying the storage capacity. In this sense, water balance can be defined as the equilibrium state in the physical system between inputs and the output modified by human interventions. Considering the socio-economic water inflow (si), the socio-economic water outflow (so), and the socio-economic water storage (ss), and so both human and natural systems, Equation (1) becomes [33]:

(ni + si) = (no + so) ± (ns + ss)(4)

The socio-economic service water inflow (si) depends on two main components: the external inflow of rivers and groundwater that enters at the neighbourhood scale and the water returned by socio-economic systems (as wastewater). The external natural water inflows consist of river water (ns_1_) and groundwater (ns_2_). Furthermore, socio-economic water inflows consist of the volume of water directly discharged from residential buildings (si_3_), and other types of buildings such as offices and/or schools (si_4_), and lost from the wastewater collection as an overflow, so by the polluted surface rainwater (si_5_) and by the infiltrated rainwater (si_6_). 

The socio-economic water outflow (so) depends directly on the water needed by the different socio-economic activities. According to the simplified approach of this research, we identified only the following sub-indicators of socio-economic water outflow: green space irrigation (so_1_), human activities such as street cleaning (so_2_), residential buildings (so_3_), and other buildings (office, school, etc.) (so_4_).

Finally, the water storage depends on groundwater storage (ns_1_) and on external neighbourhood water support (ns_2_); furthermore, socio-economic water storage relates to the water supply plant (ss_1_) and underground water storage (ss_2_).

It should be noted that in practice there are many sub-indicators, but in our simplified approach proposing a schematic overview of the urban systems, only those listed are considered. Considering all the sub-indicators, Equation (4) becomes:

[ni + (si_1_ + si_2_ + si_3_ + si_4_ + si_5_ + si_6_)] = [(no_1_ + no_2_) + (so_1_ + so_2_ + so_3_ + so_4_)] ± [(ns_1_ + ns_2_ + ss_1_ + ss_2_) (5)

The indicators and sub-indicators mentioned before are identified in the table below (Table 1):

Regarding the essential services for citizens, indicators for measuring water supply resilience can be divided into four dimensions: technic, organisational, social, and economic [61]. Several studies [60,61,62,63,64] present a large number of indicators for the resilience or vulnerability of water supply systems. Among these indicators, urban water demand or supply is frequently used as an indicator for the resilience assessment of water supply systems with regards to water balance [65,66,67].

### 3.2. Applying the “Behind the Barriers” Model to Water Management

According to the BB model [34], the water resilience assessment of a neighbourhood starts with the study of cognitive resilience which consists in making a diagnosis of the urban area based on a holistic approach. The analysis of cognitive aspects is frequently based on local quantitative or qualitative resources, such as government documents, graphs, digital data, etc.

A neighbourhood is “a part of a city with its own appearance and characterised by distinctive features which give it a degree of unity and individuality” [68]. A neighbourhood can be represented as an open and complex system characterised by exchange processes within its environment and with the external environment in continuous change and development [37].

Concerning urban issues, firstly it is necessary to identify the specificities and identity of the neighbourhood in terms of, among other characteristics, the morphological features, the urban limits, and the urban structure. Then, the neighbourhood should be located in the urban area and its environment should be characterised. For example, concerning water-related issues, it is necessary to identify the hydrogeological basin; in fact, under particular climatic scenarios the hydrogeomorphological characteristics may lead to water scarcity conditions. In parallel, it is necessary to identify potential hazards in order to then identify the vulnerability of different exposed assets (such as population, economic activities, critical infrastructures, etc.), which must be determined to understand the water-related issues and their potential consequences. Finally, cognitive resilience should also be integrated into the cognitive resilience aspects related to the management policy. Some specific regulatory tools, such as different measures and strategies, provide policies and approaches on how the crisis can be anticipated or how the neighbourhood inhabitants can participate in risk management. The goal is to know the neighbourhood needs and capacity to respond to water-related crises.

A further step towards cognitive resilience, which is a prerequisite for the application of functional resilience, is understanding how the neighbourhood system works through functional analysis (FA). The FA highlights the functional relationships between the neighbourhood components and shows both high dependencies and interdependencies between components within or outside the neighbourhood [69]. It defines the boundaries of the system, its environment, and the functions it provides [70].

Through the definition of cognitive resilience, it is then possible to assess the crisis and no-crisis scenarios. The design of specific indicators for each case study, generally accompanied by a defined criterion [71,72], is based on the implementing actions and the objectives to be achieved. In this study, in the framework of urban water management at the neighbourhood scale, “Water balance” is considered as a performance criterion, and its assessment involves three indicators: water storage, water inflow, and water outflow [31,32,33]. This performance criterion has only two levels: when water inflow is equal to the sum of water outflow and the change in water storage, i.e., Inflow = Outflow ± Changes in Storage (1), the water management in the studied neighbourhood achieves water balance, otherwise not.

Following the BB model, the second step is the assessment of the functional resilience of the neighbourhood based on the damage to its urban functions. Indeed, under water-related crises, high dependencies and interdependencies between neighbourhood components can become real issues compromising the operation of the entire city [37,69,73,74].

At the urban scale, among urban components critical infrastructure (CI) stands as an infrastructure essential for the functioning of society [75,76]; at the neighbourhood scale, the concept of major infrastructure (MI) has been developed to define those urban components which are essential for the proper operation of a neighbourhood, whose failure would have a serious impact on its inhabitants [37].

In this scenario, improving the functional resilience of a neighbourhood means increasing the neighbourhood’s ability to protect itself from its MI damage, thereby reducing its possible functional failure. The implementation of functional resilience to water-related crises can be achieved through actions and strategies that improve the security, redundancy, and stock management related to the MIs of a neighbourhood. Since MIs are essential for the proper operation of a neighbourhood, there is an important demand for services provided by them. Moreover, during a crisis, these infrastructures are usually the most vulnerable as a consequence of their complexity and dependency on each other.

Once functional resilience has been addressed, the third step is to assess correlative resilience. Studying the correlative resilience of a neighbourhood consists of analysing the link between the failure or degradation of a service provided by an MI and the acceptability of this failure or degradation. The goal is to reduce the need for such services to offer a degraded operation mode of a neighbourhood. Indeed, reducing the demand made for an MI may enable it to be kept in operation, being protected so that it can be recovered more rapidly. Thus, the correlative resilience of a neighbourhood will be possible when the correlation between MI services and the use of these services remains acceptable under crisis scenarios, yet it is necessary to analyse the measures and/or actions that allow the achievement of this goal. For example, during the lockdown forced by the COVID-19 pandemic, several buildings (such as offices or schools) were closed, modifying the needs of the neighbourhoods [77,78,79,80,81] and consequently the MI services that could operate under critical conditions [82,83].

Finally, organisational or territorial resilience expresses the capacity to mobilise the urban area outside the area directly impacted. Dependencies and interdependencies between the neighbourhood components and larger urban areas may also form an important resilience factor concerning the technical and physical dimensions of neighbourhoods. If an MI ceases to work (if it does not achieve its function), it is necessary to analyse the organisation of and the type of connections and/or relationships between the neighbourhood and its external environmental factors that can contribute to mitigating this failure as well as to recover an acceptable level of performance as soon as possible. Indeed, the territorial resilience of a neighbourhood can be defined as the capacity of a neighbourhood to mobilise larger urban scales to improve its own resilience.

To further explain the approach, the next section introduces as a case study the Part-Dieu neighbourhood in Lyon (France), which may potentially be subject to several water-related crises due to natural and/or human-made risks. Part-Dieu was also chosen because it is trying to become a more resilient neighbourhood through urban retrofit processes.

## 4. Case Study: The Water Management in the Part-Dieu Neighbourhood in Lyon

**Figure 2:**
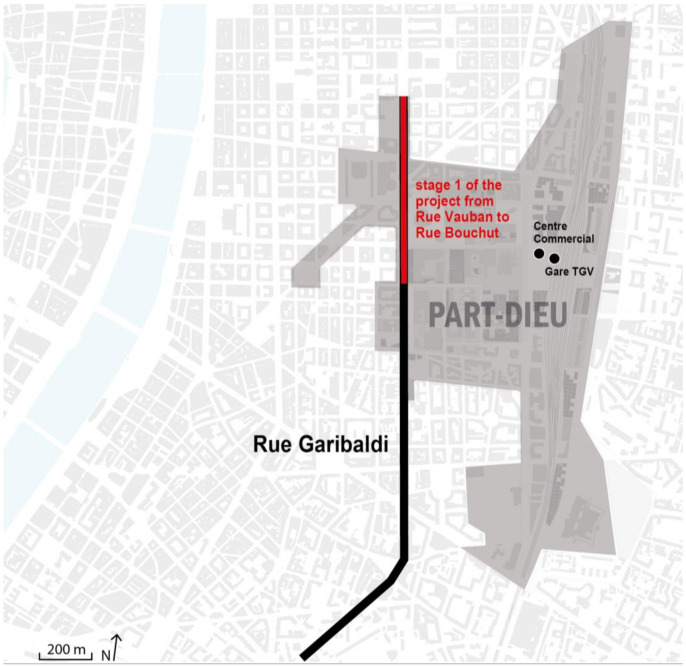
General view of Lyon Part-Dieu business district, realised by authors. Sources: Grand Lyon, La Metropole [84].

Lyon is situated at the confluence of the Rhône and Saône rivers and is the third-largest city in France. On the left bank of the Rhône, the Lyon Part-Dieu business district was designed in the 1960s and 1970s as the economic and transport centre of Europe, and today this area is trying to develop its commercial character and to improve its ecological capacity to become a more sustainable urban space [84] (see Figure 2).

In the following paragraphs, Section 4.1 shows the first (knowledge of the area, including hazard scenarios identification) and second steps (action definition) of the BB model application in terms of action performance to understand and improve the water balance state in the Part-Dieu neighbourhood. Following these results, Section 4.2 establishes the indicators and sub-indicators of action performance. After that, the discussion section presents the results of the action performance assessment through cognitive, functional, correlative, and territorial analysis.

### 4.1. Understanding Hazard Scenario and Cognitive Resilience: The State of the Art

Simplifying a complex process, water management in Lyon, as in most cities, is characterised by the presence of a water supply system that treats water from the river and then supplies it to all infrastructures, urban services, and buildings. Urban wastewater is then treated through wastewater treatment plants and then discharged into the river, while polluted rainwater is discharged directly into the river through storm sewers. The water management cycle is therefore based on a series of interdependent systems called the urban infrastructure.

This complex system in the next few years in Lyon and Part-Dieu, as in many European and other cities, could be subject to increasingly intense and frequent crises due to changes in the demand, supply, and availability of water resources. These crises could be caused by an increase in drought events due to global warming and, therefore, by a scarcity of water [85], or by the water resource quality threatened by climate change and pollution [86], or by the change in residential water demand due to the COVID-19 pandemic [15]. Furthermore, Lyon Metropole has been classified as a High Flood Risk Area (TRI) by the prefectural decree of 27 April 2012, due to the potential impacts on human health and economic activities [87]. In this context, it is evident that the water balance condition will be subject to multiple stresses in the coming years.

The traditional water infrastructures may no longer be able to respond to the increasing severity and frequency of hazards, making innovative and sustainable solutions urgently necessary for resilient water management in Lyon and the Part-Dieu district. To identify where and why possible failures might occur, following the BB model, it is necessary to study cognitive resilience aimed at identifying potential crises through a multi-risk and multi-scale approach.

**Figure 3:**
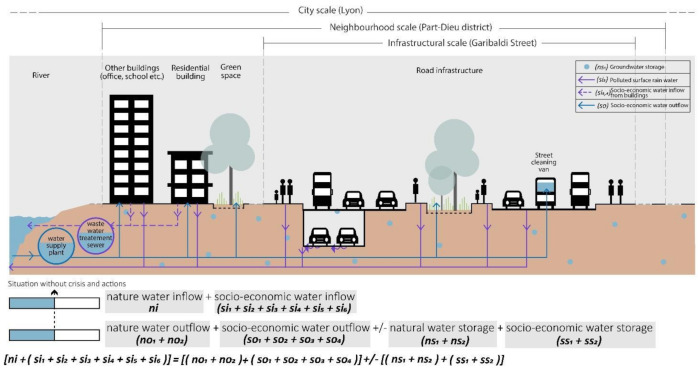
No-crisis situation, cognitive resilience in Part-Dieu District, realised by authors.

The first step is to analyse the case study area under “no crisis “ conditions, i.e., under conditions where there is a state of water balance (Equation (5), Figure 3).

Once the no-crisis situation has been investigated, potential crisis scenarios are studied, considering the case of a no-water-balance condition (Equation (6)):

[ni + (si_1_ + si_2_ + si_3_ + si_4_ + si_5_ + si_6_)] ≠ [(no_1_ + no_2_) + (so_1_ + so_2_ + so_3_ + so_4_)] ± [(ns_1_ + ns_2_ + ss_1_ + ss_2_)] (6)

This condition of a water-related crisis scenario in the case of Part-Dieu neighbourhood, Lyon could be related to a condition of water scarcity (drought scenario: DS), excess water (rainy scenario: RS), and finally a water scarcity due to changes in water demand caused by the COVID-19 pandemic (COVID-19 scenario: CS).

The first crisis scenario analysed is the drought scenario, which leads to a lack/shortage of water resources. According to several studies focusing on the Lyon environment [88,89,90], the phenomenon of urban heat islands amplifies the impacts on public health and energy consumption. As explained by the vice-president of the community in charge of urban planning and the living environment [91], the heat rises are very impactful in terms of water resources, and the risks are the subsidence of the water level as well as the levels of the rivers that run through this city. For example, on 10 august 2022, Part-Dieu district was placed in a “crisis” situation at the level of drought. Due to this crisis, the amount of water resources was not enough to supply all the citizens’ needs. As a result, it was forbidden to collect water from rivers or underground water sources; to water vegetable gardens during the day; to fill or top up swimming pools; to water lawns, green spaces, and sports fields; and to wash cars, facades, or roofs [92].

**Figure 4:**
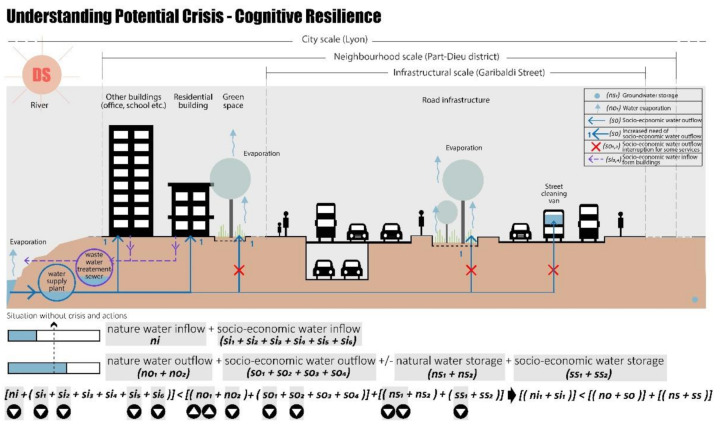
Drought scenario, cognitive resilience in Part-Dieu district, realised by authors.

During the DS, decreased rainwater (ni) and increased evaporation (no_1_) reduce total natural water storage (ns) and thus total water service inflow (si). Meanwhile, water service outflow demand (so) for most of the equipment increases. As temperatures rise and water shortages become more acute, demand will change [93,94,95,96,97], more water will be needed for irrigating green spaces (Figure 4, purple lines (1). These changes, in Part-Dieu district, can create a crisis related to a condition of water scarcity in which the water inflow is less than water outflow plus water storage (Equation (7)).

(ni + si) < (no + so) + (ns + ss)(7)

The water supply may thus be inadequate for critical urban infrastructures, referring specifically to residential buildings in this study, but also for non-vital but important city services, such as green space irrigation or street cleaning. Furthermore, the rise in temperature can create a condition of land drought due to the grave reduction in natural water storage (ns) exacerbating the crisis scenario.

In summary, the drought-season crises can be related to:

- A decrease in water inflow caused by a decrease in water resources.
- An increase in water outflow caused by an increase in water demand.
- A decrease in water storage caused by a decrease in water resources.

At the same time, in the Lyon Metropolis, flood risks are increasingly alarming managers and citizens and becoming a danger that could spread. The floods in Lyon can be generated by heavy oceanic precipitation, Mediterranean rainfall episodes of high intensity, or stormy episodes with high intensity [98]. A recent flooding event occurred on 17 August 2022, after long weeks of high temperatures, even heat waves and drought periods. In many urban areas, including Part-Dieu, huge puddles of water formed, and some streets were even completely flooded [99].

**Figure 5:**
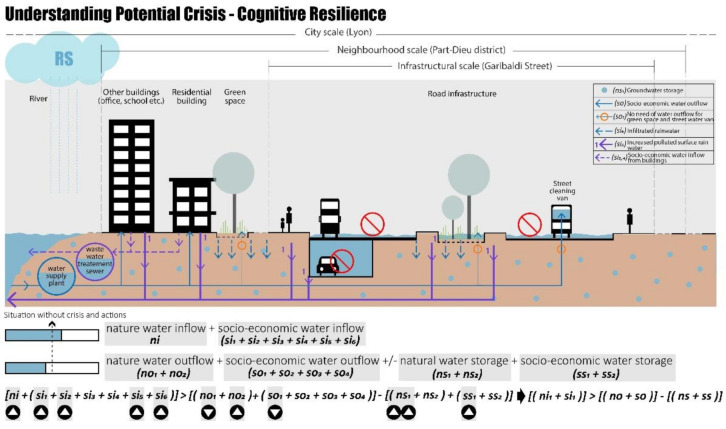
Rainy scenario, cognitive resilience in Part-Dieu District, realised by authors.

This type of event can be simplified and is shown in Figure 5. During the rainy scenario (RS), the precipitation rainwater (ni) increases, together with an increase in river water (si_1_), groundwater (si_2_), polluted surface rainwater (si_5_), and infiltrated rainwater (si_6_). These conditions create an increase in water inflow both natural and socio-economic (ni + si). Meanwhile, the water outflow decreases, and the water evaporation (no_1_) together with the water demand for green spaces (so_1_) and for street cleaning (so_2_) are lower. These conditions could decrease the capacity of natural water storage (ns) and cause urban flooding, exacerbating the water runoff (no_2_), affecting, directly or indirectly, the performance of urban infrastructures, such as roads and green spaces.

These changes in sub-indicators, in Part-Dieu district, can create a crisis in which the water inflow is less than water outflow less the water storage (Equation (8)):

(ni + si) > (no + so) − (ns + ss)(8)

In summary, the rainy-season crisis is related to:

- An increase in water inflow both natural and socio-economic.
- A decrease in water outflow.
- An increase in water storage, and thus a decrease in water storage capacity.

**Figure 6:**
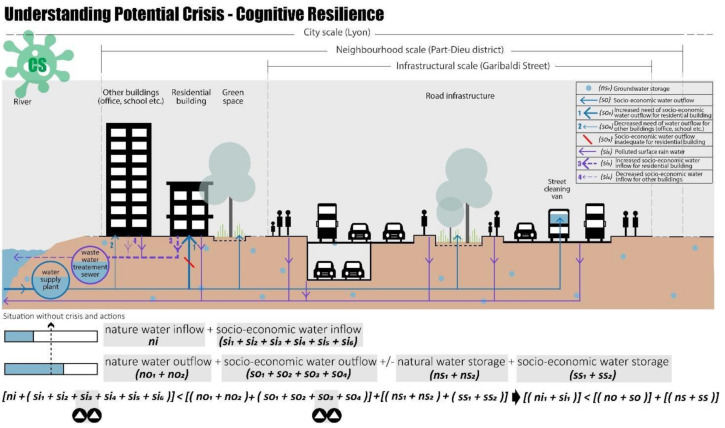
Cognitive resilience in Part-Dieu district, COVID-19 scenario, realised by authors.

Finally, the last crisis scenario can be identified as the water scarcity caused by a change in water demand due to the COVID-19 pandemic (Figure 6). During these periods, an increase in daily household water demand was identified [77,78,79,80,81]. According to the data by Insee [100], in France, one-third of companies closed during the first containment for 57 days, people remained at home, and the demand for water resources for non-residential buildings was reduced.

The COVID-19 scenario in the Part-Dieu neighbourhood could create a crisis related to an increase in the water demand for residential buildings (ss_3_) that could not be covered by the water inflow, also considering the water storage, resulting in a shortage of residential water use (Equation (9)):

(ni + si) < (no + so) + (ns + ss)(9)

Considering these crisis scenarios, as mentioned in Section 3.2, to assess resilience it is necessary to use specific indicators for each case study based on the implementing actions and the objectives to be achieved; in this case study, the action objective is the water balance of Part-Dieu district.

### 4.2. Identification of Resilience Actions

To apply the BB method to a real case study and to deepen its contribution, the urban retrofitting project of Garibaldi Street is considered an action for improving functional resilience. Garibaldi Street is one of the major strategic arterial roads of Lyon; it was designed in the 1960s as an “urban motorway”, as a result of the changing exigencies of contemporary life, and has been subject to continuous renovation since the 1990s [84].

The analysed project area consists of 2.6 km which, through the reorganisation of road, cycle, and pedestrian traffic, creates a large, vegetated area to improve the hydraulic management of rainwater, treat rainwater, prevent urban heat islands, and, finally, create new public spaces. The first road section, between Vauban and Bouchut, was already completed in 2016 (see Figure 2). The project consists of tree-based rainwater recycling and can be considered a functional action, aimed at reducing the impacts of extreme weather [101]. To maximise risk management combined with ecological benefits, the project integrates multiple design solutions, including different levels of service, through measures of hard engineering and soft engineering.

The identified actions linked to correlative and territorial resilience are, respectively, reducing water supply to some buildings and using it for others and increasing water resource inflow using external neighbourhood (or city) water resources. As these two measures are based on assumptions about potential future disasters, they have been implemented considering long-term scenarios.

However, as explained above, this study also focuses on the increase in potential hazards, considering different spatial and temporal scales. The design measures, following the BB method, will first be evaluated within the “barrier” framework to analyse functional resilience and then “Behind the Barriers” integrating correlative and territorial resilience.

#### 4.2.1. Barriers Approach—Functional Resilience

**Figure 7:**
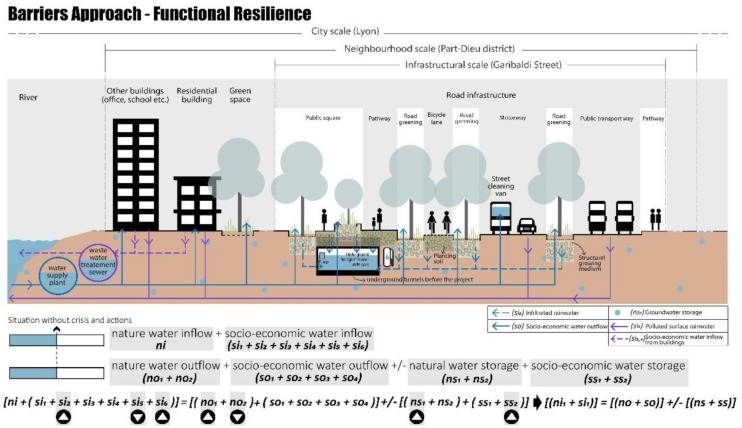
Inside the “barrier”, functional resilience Garibaldi project, no-crisis scenario (NCS), realised by authors, adapted from TDAG [101].

Functional resilience aims at improving management through changes in internal material objects and technical urban systems. The retrofitting project on Garibaldi Street focuses on both the problem of flooding (corresponding to the rainy scenario) and water scarcity (drought scenario), by using innovative systems for water storage and reuse. As shown in Figure 7, the water is managed through multiple measures [102]:

- The runoff (from the roads, pedestrians, bicycles, and public transport) is reduced, the water becomes clean after being infiltrated using sustainable materials, and then it can be collected in an old underpass that has been retrofitted as underground water storage. The sustainable materials, planting soil, and structural growing medium are used for green space and permeable pavement, allowing an increase in groundwater storage (sub-indicators involved: si_2_, si_5_, si_6_, no_1_, no_2_, ns_1_, ss_2_) and reducing the outdoor temperature [103];
- The collected clean water can be reused for green space irrigation and/or street cleaning, especially during the drought season when the water demand increases;
- In the case of heavy storms, the water can also be controlled and conveyed to the existing combined sewer by using the pump in the storage.

These functional actions, in the condition of the no-crisis scenario (NCS), reduce the total water service outflow (so), and thus the water saved can be stored in the new underground storage basin (ss_2_).

**Figure 8:**
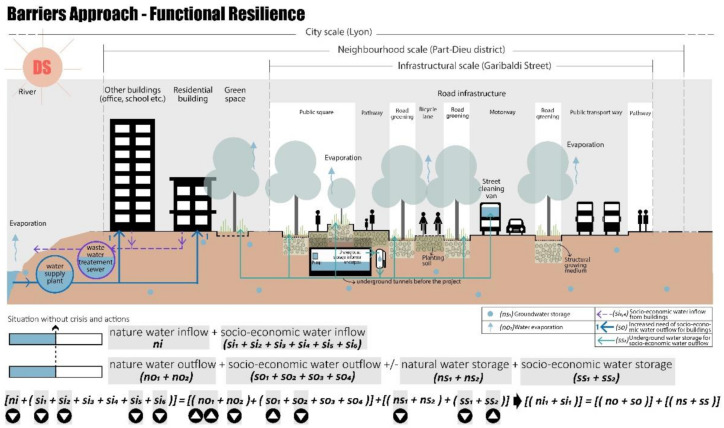
Inside the “barrier”, functional resilience drought scenario, realised by authors, adapted from TDAG [101].

Moreover, under crisis conditions these solutions could solve the problems related to the drought and rainy scenarios. In the case of a drought (DS), even though water inflow heavily decreases due to reduced precipitation water (ni) and river water (si_2_), the water storage (ns_1_, ss_2_), which increased during the normal situation thanks to the permeability of the new soil surfaces, could be used for socio-economic water outflow as shown in Figure 8.

**Figure 9:**
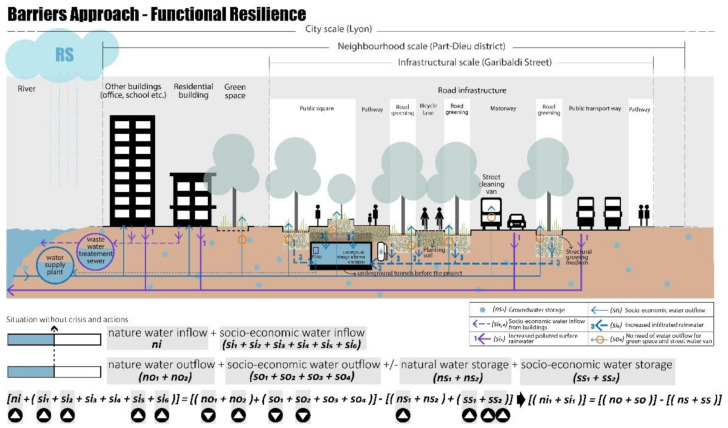
Inside the “barrier”, functional resilience rainy scenario, realised by authors, adapted from TDAG [101].

As shown in Figure 9, in the case of rainy scenarios (RS), the excess precipitation water (ni) can be collected in the new underground water storage basin (ss_2_), preventing urban floods. Thus, also in this crisis scenario the socio-economic water inflow increases thanks to the new design solutions (sub-indicators involved: si_2_, si_5_, si_6_, no_1_, no_2_, so_1_, so_2_, ns_1_, ss_2_, ns_1_).

#### 4.2.2. Behind the Barriers Approach—Correlative and Territorial Resilience

Following the BB theory, going “Behind the Barriers” means going behind the spatial–temporal barriers of the study system to achieve the water balance in the Part-Dieu neighbourhood and without considering the retrofit project of Rue Garibaldi.

Considering the neighbourhood scale and the COVID-19 scenario, the water balance can be achieved through correlative actions. During the pandemic period, to prevent the spread of the virus, the French government imposed a lockdown and permitted smart working; these measures allowed a significant reduction in water outflow demand by offices and schools (si_4_), and consequently in the associated water outflow (so_4_).

**Figure 10:**
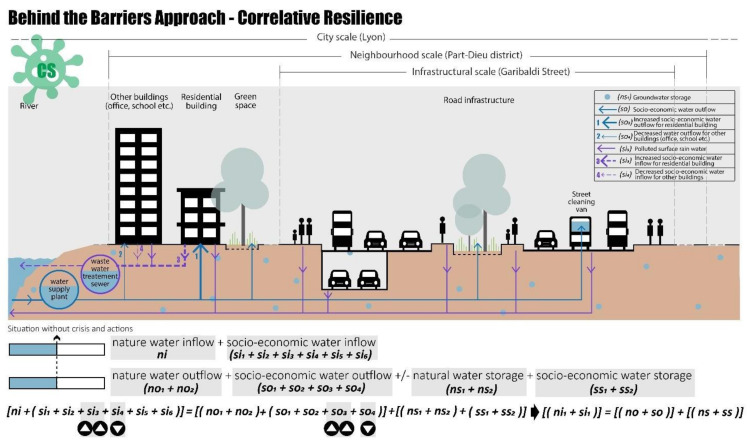
Behind the “barrier”, correlative resilience in COVID-19 scenario, realised by authors.

To achieve water balance, the higher water demand by residential buildings could be solved using the saved water from the other types of buildings (si_3_), as shown in Figure 10.

**Figure 11:**
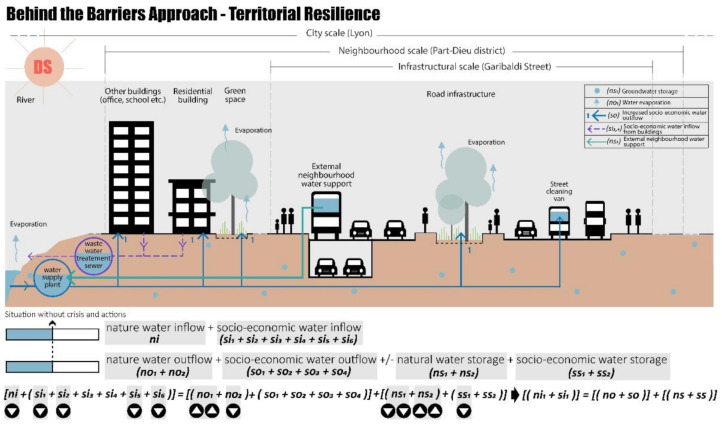
Behind the “barrier”, territorial resilience drought scenario, realised by authors.

At the same time, considering the city scale and the drought scenario (DS), the water balance can be achieved through territorial actions, as shown in Figure 11. To avoid the pressure on the Part-Dieu neighbourhood, considering larger spatial scales, the required water can be taken from other neighbourhoods by increasing the natural water inflow, considering the sub-indicator of the external neighbourhood water support (ns_2_) in terms of the new availability of natural water storage. Certainly, in the case of the drought scenario many areas will have a low water inflow, but a city-scale crisis is certainly more complex to control.

## 5. Discussion

This study focused on the application of the “Behind the Barriers” model to understand how applying resilience actions can improve the capacity of urban systems in the case of crisis scenarios. In particular, the case study addressed the problem of water balance failure, a condition that can lead to several problems by impacting the performance of cities.

As detailed in Section 4, the object of the case study was to assess the performance of designed measures for achieving water balance at the neighbourhood scale. The contribution, following the BB model, was structured in four methodological steps:

1. Analyse cognitive resilience to understand the current state scenario and potential crisis scenarios;
2. Analyse functional resilience to understand how the retrofitting project of Rue Garibaldi and its functional actions could improve the performance of the water system in case of drought and flood scenarios;
3. Analyse correlative resilience to understand how correlative actions could improve the performance of the water system in case of a drought scenario;
4. Analyse territorial resilience to understand how territorial actions could improve the performance of the water system in case of a COVID-19 scenario.

**Figure 12:**
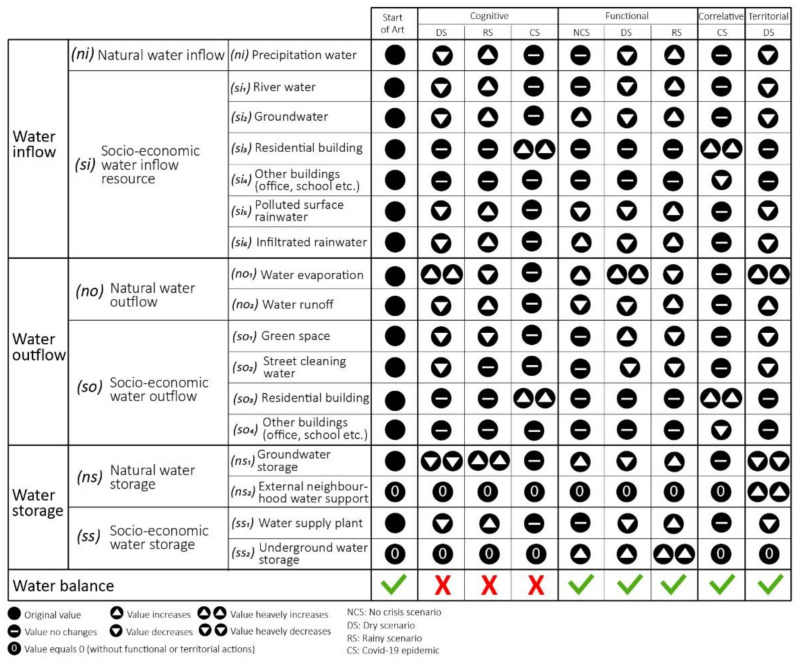
Changes in indicators and sub-indicators in the Part-Dieu neighbourhood in Lyon, after applying resilience actions.

All indicators and sub-indicators involved to achieve the condition of water balance at the neighbourhood scale are indicated in Figure 12. This figure compares the changes in each sub-indicator after resilience actions and shows how performing these actions (functional, correlative, and territorial) can also achieve the water balance condition during crisis scenarios (Figure 12). When water inflow is equal to the sum of water outflow and the change in water storage, i.e., Inflow = Outflow ± Changes in Storage (1), the performance level of water management is achieving water balance (green in Figure 12), otherwise not (red in Figure 12). As shown in the figure, the resilience actions can achieve the condition of water balance in the Part-Dieu neighbourhood in Lyon in the case of a crisis (drought, flood, and COVID-19 scenarios).

The application of the case study thus highlighted the need to develop indicators to assess the performance of implemented actions within a framework of structural and global resilience to support decision makers. Furthermore, it was highlighted that the implementation of resilience requires an understanding of urban systems as complex and dynamic systems, as well as the enhancement of crisis prevention and management perspectives with a dynamic and multi-risk approach.

Since resilience has been studied for urban disasters, many theories have emerged on the research approach. Although this paper focuses on water balance in the Part-Dieu neighbourhood, as the “Behind the Barriers” theory is a multidisciplinary, transversal resilience theory aiming at adaptation capacity, the approach can be considered suitable also in other types of urban disaster research and for the management of other types of urban systems.

## 6. Conclusions

Choosing the correct approach to studying resilience is difficult because of its complexity and the fact that its evaluation is highly sensitive to contextual factors. These contextual factors, irrespective of whether they are urban, social, or environmental, are often responsible for the failure to develop generic methods. Whenever studying local resilience systems is concerned, the initial difficulty resides in defining the system’s limits and the elements it contains, objectives, and resources available.

For these reasons, the neighbourhood scale is an important scale when designing resilient urban planning. The “Behind the Barriers” conceptual model is developed for understanding and improving urban resilience thanks to four types of resilience actions: cognitive, functional, correlative, and territorial. The evaluation of the performance of actions to achieve the resilient state is assessed based on indicators and sub-indicators. As such, the global approach to resilience based on the “Behind the Barriers” conceptual model is materialised on the local neighbourhood scale by flow studies.

This study applies the BB model to the Part-Dieu neighbourhood in Lyon to study the water balance condition. Through experimentation on a real case study, the model highlights its potential as a strategic tool to support decision-making processes in implementing urban resilience. Indeed, the nature of urban systems is highly complex, and to orient design projects, make urban infrastructures resilient, and ensure the city’s performance, it is necessary to integrate multiple aspects.

## Figures and Tables

**Table 1:** Indicators and sub-indicators involved in water balance.

Indicators		Sub-Indicators
Water inflow	Natural water inflow (ni)	Precipitation water (ni)
	Socio-economic water inflow resource (si)	River water (si_1_)
		Groundwater (si_2_)
		Residential building (si_3_)
		Other buildings (office, school, etc.) (si_4_)
		Polluted surface rainwater (si_5_)
		Infiltrated rainwater (si_6_)
Water outflow	Natural water outflow (no)	Water evaporation (no_1_)
		Water runoff (no_2_)
	Socio-economic water outflow (so)	Green space irrigation (so_1_)
		Street cleaning water (so_2_)
		Residential building (so_3_)
		Other buildings (office, school, etc.) (so_4_)
Water storage	Natural water storage (ns)	Groundwater storage (ns_1_)
		External neighbourhood water support (ns_2_)
	Socio-economic water storage (ss)	Water supply plant (ss_1_)
		Underground water storage (ss_2_)

## Data Availability

Not applicable.

## References

[1] Parry M.L., Canziani O.F., Palutikof J.P., van der Linden P.J., Hanson C.E. (2007). 2007: Technical Summary. Climate Change 2007: Impacts, Adaptation and Vulnerability: Contribution of Working Group II to the Fourth Assessment Report of the Intergovernmental Panel on Climate Change.

[2] Lamarre D. (2008). Climat et Risques: Changements D’Approches.

[3] IPCC. https://www.ipcc.ch/report/sixth-assessment-report-working-group-ii/.

[4] Zevenbergen C., Cashman A., Evelpidou N., Pasche E., Garvin S., Ashley R. (2010). Urban Flood Management.

[5] Serre D., Barroca B., Laganier R. (2012). Resilience and Urban Risk Management.

[6] United Nation. https://www.un.org/development/desa/publications/2018-revision-of-world-urbanization-prospects.html.

[7] McDonald R.I., Weber K., Padowski J., Flörke M., Schneider C., Green P.A., Gleeson T., Eckman S., Lehner B., Balk D. (2014). Water on an urban planet: Urbanization and the reach of urban water infrastructure. Glob. Environ. Change.

[8] Romano G., Salvati N., Guerrini A. (2016). An Empirical Analysis of the Determinants of Water Demand in Italy. J. Clean. Prod..

[9] Vörösmarty C.J., Green P.J., Salisbury J., Lammers R.B. (2000). Global water resources: Vulnerability from climate change and population growth. Science.

[10] Haddeland I., Biemans H., Flörke M., Hanasaki N., Stacke T., Tessler Z.D., Wada Y. (2013). Multimodel Estimate of Global Water Resources Affected by Human Interventions and Climate Change. AGU Fall Meeting Abstracts.

[11] Hagemann S., Chen C., Clark D., Folwell S., Gosling S.N., Haddeland I., Hannasaki N., Heinke J., Ludwig F., Voss F. (2013). Climate change impact on available water resources obtained using multiple global climate and hydrology models. Earth Syst. Dyn..

[12] Cramer W., Guiot J., Fader M., Garrabou J., Gattuso J.-P., Iglesias A., Lange M.A., Lionello P., Llasat M.C., Paz S. (2018). Climate change and interconnected risks to sustainable development in the Mediterranean. Nat. Clim. Chang..

[13] Heidari H., Arabi M., Warziniack T., Sharvelle S. (2021). Effects of Urban Development Patterns on Municipal Water Shortage. Front. Water.

[14] United Nation. https://www.unwater.org/equitable-access-to-water-and-sanitation-is-still-a-challenge-for-europe/.

[15] Mukhtarov F., Papyrakis E., Rieger M. (2022). COVID-19 and water. COVID-19 and International Development.

[16] Coombes P., Mitchell G., Wong T.H.F. (2006). Urban water harvesting and reuse. Australian Runoff Quality: A Guide to Water Sensitive Urban Design.

[17] Jiri M., Blanca J.C., Mohammed K., Per-Arne M., Joel G., Bernard C. (2007). Urban Water Cycle Process and Interactions.

[18] Hardy M.J., Kuczera G., Coombes P.J. (2005). Integrated urban water cycle management: The Urban Cycle model. Water Sci. Technol..

[19] Marlow D.R., Moglia M., Cook S., Beale D.J. (2013). Towards sustainable urban water management: A critical reassessment. Water Res..

[20] Ma X.C., Xue X., González-Mejía A., Garland J., Cashdollar J. (2015). Sustainable water systems for the city of tomorrow—A conceptual framework. Sustainability.

[21] Piratla K.R., Goverdhanam S. (2015). Decentralized Water Systems for Sustainable and Reliable Supply. Procedia Eng..

[22] Pearlmutter D., Pucher B., Calheiros C.S.C., Hoffmann K.A., Aicher A., Pinho P., Stracqualursi A., Korolova A., Pobric A., Galvão A. (2021). Closing Water Cycles in the Built Environment through Nature-Based Solutions: The Contribution of Vertical Greening Systems and Green Roofs. Water.

[23] Folke C., Carpenter S.R., Walker B., Scheffer M., Chapin T., Rockström J. (2010). Resilience thinking: Integrating resilience, adaptability and transformability. Ecol. Soc..

[24] Miller F., Osbahr H., Boyd E., Thomalla F., Bharwani S., Ziervogel G., Walker B., Birkmann J., Van der Leeuw S., Rockström J. (2010). Resilience and vulnerability: Complementary or conflicting concepts?. Ecol. Soc..

[25] Lei Y., Wang J., Yue Y., Zhou H., Yin W. (2014). Rethinking the relationships of vulnerability, resilience, and adaptation from a disaster risk perspective. Nat. Hazards.

[26] Proag V. (2014). The concept of vulnerability and resilience. Procedia Econ. Financ..

[27] Meerow S., Newell J.P., Stults M. (2016). Defining urban resilience: A review. Landsc. Urban Plan..

[28] Falkenmark M., Galaz V. (2007). Agriculture, Water and Ecosystems, Policy Brief No. 6.

[29] Imbulana N., Manoharan S. (2020). Hydrological and water balance studies to evaluate options for climate resilience in smallholder irrigation systems in Sri Lanka. Water Policy.

[30] Liu L., Jensen M.B., Zhang X. (2019). Urban Water Management in Beijing and Copenhagen: Sustainability, Climate Resilience, and the Local Water Balance. Greening China’s Urban Governance.

[31] Sokolov A.A., Chapman T.G. (1974). Methods for Water Balance Computations.

[32] de Ridder N.A., Boonstra J., Ritzema H.P. (1994). Analysis of Water Balances. Drainage Principles and Application.

[33] European Commission. https://op.europa.eu/en/publication-detail/-/publication/7d148604-faf0-11e5-b713-01aa75ed71a1/language-en.

[34] Barroca B., Serre D. (2013). Behind the Barriers: A Resilience Conceptual Model. Surv. Perspect. Integr. Environ. Soc..

[35] Bahri A. (2012). Integrated Urban Water Management.

[36] Folke C. (2006). Resilience: The emergence of a perspective for social-ecological systems analyses. Glob. Environ. Change.

[37] Balsells M., Barroca B., Amdal J.R., Diab Y., Becue V., Serre D. (2013). Analysing urban resilience through alternative stormwater management options: Application of the conceptual Spatial Decision Support System model at the neighbourhood scale. Water Sci. Technol..

[38] Weichselgartner J., Kelman I. (2014). Challenges and opportunities for building urban resilience. J. Facul. Architectur..

[39] Pearson L.J., Newton P.W., Roberts P. (2014). Introduction to the magic and practice of resilient, sustainable cities. Resilient Sustainable Cities.

[40] Gersonius B., Nasruddin F., Ashley R., Jeuken A., Pathirana A., Zevenbergen C. (2012). Developing the evidence base for mainstreaming adaptation of stormwater systems to climate change. Water Res..

[41] Bacchin T., Ashley R., Veerbeek W., Pont M.B. A multi-scale approach in the planning and design of water sensitive environments. Proceedings of the 8ème Conférence Internationale sur les Techniques et Stratégies Durables pour la Gestion des Eaux Urbaines par temps de pluie/8th International Conference on Planning and Technologies for Sustainable Management of Water in the City.

[42] RESILIS Project. www.resilis.fr/en.

[43] Heinzlef C., Barroca B., Leone M., Serre D. (2022). Urban resilience operationalization issues in climate risk management: A review. Int. J. Disaster Risk Reduct..

[44] Ahern J. (2011). From fail-safe to safe-to-fail: Sustainability and resilience in the new urban world. Landsc. Urban Plan..

[45] Sajaloli B., Servain-Courant S., Dournel S., Andrieu D. (2011). L’inscription paysagère du risque d’inondation dans les politiques urbaines des agglomérations ligériennes, proposition d’un marqueur de résilience spatiale. Rev. Géographique de l’Est.

[46] Lhomme S. (2012). Les Réseaux Techniques Comme Vecteur de Propagation des Risques en Milieu Urbain-Une Contribution Théorique et Pratique à L’analyse de la Résilience Urbaine. Ph.D. Dissertation.

[47] Barroca B., Clemente M.F., D’Ambrosio V., Bonciani B., Bordato L., Giovene di Girasole E. (2021). Resilienza funzionale dei sistemi portuali e strategie per il progetto climate proof. Dialoghi tra Porto e Città Nell’epoca Della Globalizzazione.

[48] FloodProBE. https://www.floodprobe.eu/.

[49] SMARTeST Project. https://www.floodguidance.co.uk/smartest-project/.

[50] De Graff R., Roeffen B., Czapiewska K.M., Dal Bo Zanon B., Lindemans W., Escarameia M., Walliman N.S.R., Zevenbergen C., Klijn F., Schweckendiek T. (2012). The effectiveness of flood proofing vulnerable hotspots to improve urban flood resilience. Comprehensive flood risk management: Research for policy and practice. 2nd European conference on floodrisk management FLOODrisk2012.

[51] Zevenbergen C., Veerbeek W., Gersonius B., Van Herk S. (2008). Challenges in urban flood management: Travelling across spatial and temporal scales. J. Flood Risk Manag..

[52] Howe C.A., Butterworth J., Smout I.K., Duffy A.M., Vairavamoorthy K. (2011). Sustainable Water Management in the City of the Future: Findings from the SWITCH Project 2006–2011.

[53] Johannessen Å., Wamsler C. (2017). What does resilience mean for urban water services?. Ecol. Soc..

[54] Pizzo B. (2015). Problematizing resilience: Implications for planning theory and practice. Cities.

[55] Sterk M., van de Leemput I.A., Peeters E.T. (2017). How to conceptualize and operationalize resilience in socio-ecological systems?. Curr. Opin. Environ. Sustain..

[56] Gonzva M. (2017). Résilience des Systèmes de Transport Guidé en Milieu Urbain: Approche Quantitative des Perturbations et Stratégies de Gestion. Ph.D. Dissertation.

[57] Gonzva M., Barroca B. Improving urban infrastructures resilience using conceptual models: Application of the “Behind the Barriers” model to the flooding of a rail transport system. Proceedings of the 7th Resilience Engineering Association Symposium.

[58] Barroca B., Serre D., Youssef D. (2012). Le concept de résilience à l’épreuve du génie urbain. VertigO—La Rev. Électron. Sci. L’Environ..

[59] Beraud H. (2013). Initier la Résilience du Service de Gestion des Déchets aux Catastrophes Naturelles: Le cas des Territoires Urbains et de L’inondation. Ph.D. Dissertation.

[60] Sebesvari Z., Renaud F.G., Haas S., Tessler Z., Hagenlocher M., Kloos J., Szabo S., Tejedor A., Kuenzer C. (2016). A review of vulnerability indicators for deltaic social–ecological systems. Sustain. Sci..

[61] Balaei B., Wilkinson S., Potangaroa R., Hassani N., Alavi-Shoshtari M. (2018). Developing a framework for measuring water supply resilience. Nat. Hazards Rev..

[62] Balaei B., Wilkinson S., Potangaroa R., McFarlane P. (2020). Investigating the technical dimension of water supply resilience to disasters. Sustain. Cities Soc..

[63] Plummer R., de Loë R., Armitage D. (2012). A systematic review of water vulnerability assessment tools. Water Resour. Manag..

[64] Baghersad M., Wilkinson S., Khatibi H. (2021). Comprehensive indicator bank for resilience of water supply systems. Adv. Civ. Eng..

[65] Li Y., Lence B.J. (2007). Estimating resilience for water resources systems. Water Resour. Res..

[66] Ghosn M., Dueñas-Osorio L., Frangopol D.M., McAllister T.P., Bocchini P., Manuel L., Ellingwood B.R., Arangio S., Bontempi F., Shah M. (2016). Performance Indicators for Structural Systems and Infrastructure Networks. J. Struct. Eng..

[67] Zhan X., Meng F., Liu S., Fu G. (2020). Comparing performance indicators for assessing and building resilient water distribution systems. J. Water Resour. Plan. Manag..

[68] Merlin F., Choay F. (2005). Dictionnaire de L’Urbanisme et de L’Amenagement.

[69] Serre D. (2016). Advanced methodology for risk and vulnerability assessment of interdependency of critical infrastructure in respect to urban floods. E3S Web Conf..

[70] Zwingelstein G. (1996). La Maintenance basée Sur la Fiabilité: Guide Pratique D’Application de la RCM.

[71] Yang Z., Barroca B., Bony-Dandrieux A., Dolidon H. (2022). Resilience Indicator of Urban Transport Infrastructure: A Review on Current Approaches. Infrastructures.

[72] Yang Z., Barroca B., Weppe A., Bony-Dandrieux A., Laffréchine K., Daclin N., November V., Kamissoko D., Benaben F., Dolidon H. (2023). Indicator-based resilience assessment for critical infrastructures—A review. Saf. Sci..

[73] Lhomme S., Serre D., Diab Y., Laganier R., Laganier R. (2013). Urban technical networks resilience assessment. Resilience and Urban Risk Management.

[74] Serre D., Heinzlef C. (2018). Assessing and mapping urban resilience to floods with respect to cascading effects through critical infrastructure networks. Int. J. Disaster Risk Reduct..

[75] EUR-Lex. https://eur-lex.europa.eu/legal-content/EN/TXT/?uri=legissum%3Al33259.

[76] Brown G., Carlyle M., Salmerón J., Wood K. (2006). Defending critical infrastructure. Interfaces.

[77] Kalbusch A., Henning E., Brikalski M.P., de Luca F.V., Konrath A.C. (2020). Impact of coronavirus (COVID-19) spread-prevention actions on urban water consumption. Resour. Conserv. Recycl..

[78] Cahill J., Hoolohan C., Lawson R., Browne A.L. (2022). COVID-19 and water demand: A review of literature and research evidence. Wiley Interdiscip. Rev. Water.

[79] Dzimińska P., Drzewiecki S., Ruman M., Kosek K., Mikołajewski K., Licznar P. (2021). The Use of Cluster Analysis to Evaluate the Impact of COVID-19 Pandemic on Daily Water Demand Patterns. Sustainability.

[80] Kazak J.K., Szewranski S., Pilawka T., Tokarczyk-Dorociak K., Janiak K., Swiader M. (2021). Changes in water demand patterns in a European city due to restrictions caused by the COVID-19 pandemic. Desalination Water Treat..

[81] Lüdtke D.U., Luetkemeier R., Schneemann M., Liehr S. (2021). Increase in Daily Household Water Demand during the First Wave of the COVID-19 Pandemic in Germany. Water.

[82] Pesantez J.E., Alghamdi F., Sabu S., Mahinthakumar G., Berglund E.Z. (2021). Using a digital twin to explore water infrastructure impacts during the COVID-19 pandemic. Sustain. Cities Soc..

[83] Cooley H., Gleick P., Abraham S., Cai W. (2020). Water and the COVID-19 Pandemic: Impacts on Municipal Water Demand.

[84] Grand Lyon, la Métropole. https://www.grandlyon.com/actions/lyon-rue-garibaldi.html.

[85] L’agence de L’eau Rhône Méditerranée Corse. https://www.eaurmc.fr/jcms/vmr_35758/fr/l-adaptation-au-changement-climatique?cid=vmr_35721&portal=cbl_7386.

[86] GrandLyon. https://www.grandlyon.com/fileadmin/user_upload/media/pdf/espace-presse/dp/2021/20211207_dp_politique-eau.pdf.

[87] Préfet de la Région Auvergne-Rhône-Alpes. https://www.auvergne-rhone-alpes.developpement-durable.gouv.fr/consultation-du-public-sur-la-gestion-de-l-eau-et-a14891.html.

[88] Maillard P., David F., Dechesne M., Bailly J.B., Lesueur E. (2014). Caractérisation des îlots de chaleur urbains et test d’une solution d’humidification de chaussée dans le quartier de la Part-Dieu à Lyon. Tech. Sci. Méthodes.

[89] Renard F., Alonso L. (2017). La combinaison de l’image satellitaire avec les données citoyennes pour la mesure de l’îlot de chaleur urbain. Premiers résultats sur la métropole de Lyon. Ingéniérie Des Syst. D’Inf..

[90] Champiat C. (2009). Identifier les îlots de chaleur urbains pour réduire l’impact sanitaire des vagues de chaleur. Environ. Risques St..

[91] Toutlyon. https://www.le-tout-lyon.fr/canicule-comment-la-metropole-de-lyon-lutte-contre-les-ilots-de-chaleur-109808.html.

[92] Rue89Lyon. https://www.rue89lyon.fr/2022/08/17/secheresse-quelles-nouvelles-restrictions-lyon-rhone/.

[93] Wilhite D.A. (2005). Drought and Water Crises.

[94] Calow R.C., MacDonald A.M., Nicol A.L., Robins N.S. (2010). Ground water security and drought in Africa: Linking availability, access, and demand. Groundwater.

[95] Mancosu N., Snyder R.L., Kyriakakis G., Spano D. (2015). Water Scarcity and Future Challenges for Food Production. Water.

[96] Mehran A., Mazdiyasni O., AghaKouchak A. (2015). A hybrid framework for assessing socioeconomic drought: Linking climate variability, local resilience, and demand. J. Geophys. Res. Atmos..

[97] Vargas J., Paneque P. (2019). Challenges for the Integration of Water Resource and Drought-Risk Management in Spain. Sustainability.

[98] Préfet du Rhône. https://www.rhone.gouv.fr/Politiques-publiques/Securite-et-protection-de-la-population/La-securite-civile/Les-risques-majeurs/Les-risques-majeurs-dans-le-Rhone/Le-Dossier-Departemental-sur-les-Risques-Majeurs/Risque-d-inondation/Les-inondations-dans-le-Rhone.

[99] ActuLyon. https://actu.fr/auvergne-rhone-alpes/lyon_69123/des-orages-inattendus-eclatent-a-lyon-des-inondations-a-prevoir_53369323.html.

[100] Insee. https://www.insee.fr/fr/statistiques/4994488.

[101] TDAG—Trees & Design Action Group. https://www.tdag.org.uk/casestudies/building-local-identities-through-tree-diversification.

[102] Graie. http://www.graie.org/graie/BaseDonneesTA/07_69_Lyon6_Garibaldi.pdf.

[103] Wang J., Meng Q., Tan K., Zhang L., Zhang Y. (2018). Experimental investigation on the influence of evaporative cooling of permeable pavements on outdoor thermal environment. Build. Environ..

